# Authors’ Reply: Accelerometer-Based Step Counts May Be Confounded by Repetitive Motor Behaviors in Adults With Autism Spectrum Disorder

**DOI:** 10.2196/113321

**Published:** 2026-10-30

**Authors:** Annabel Nunez-Gaunaurd, Melissa M Tovin, Melissa Hale, Tan Li, Anjana Bhat

**Affiliations:** 1 Department of Physical Therapy, Nicole Wertheim College of Nursing & Health Sciences, Florida International University, 11200 SW 8th St, AHC3-424, Miami, FL, 33199, United States, 1 305-348-2265; 2 Department of Physical Therapy, Miller School of Medicine, University of Miami, Coral Gables, FL, United States; 3 Child and Family Services, College of Arts and Sciences, University of Miami, Coral Gables, FL, United States; 4 Biostatistics, Robert Stempel College of Public Health and Social Work, Florida International University, Miami, FL, United States; 5 Department of Physical Therapy, College of Health Sciences, University of Delaware, Newark, DE, United States

**Keywords:** autism spectrum disorder, physical activity, co-participation, family support, autism in adulthood, telehealth, wearable devices, physical function, remote assessment, health disparities, minority health, adult autism, protocol study, cross-sectional design, patient-reported outcomes, motor impairments, rehabilitation, digital health

We thank the authors of the letter to the editor [1] for their thoughtful comments regarding our protocol paper, “Remote Assessment of Physical Function and Physical Activity in Adults With Autism Spectrum Disorder: Protocol for Feasibility, Validity, and Reliability Study” [2]. We appreciate their interest in the methodological considerations surrounding accelerometer-derived step counts in adults with autism spectrum disorder (ASD).

The authors raise an important concern regarding the potential influence of repetitive motor behaviors, including stereotypic movements such as hand flapping, rocking, and pacing, on accelerometer-based step count estimates. We agree that consumer-grade wearable devices are primarily developed and validated in neurotypical populations and that movement characteristics unique to individuals with ASD may affect measurement accuracy [3,4]. In particular, wrist-worn accelerometers may not consistently distinguish ambulatory activity from certain repetitive movements, potentially leading to overestimation of step counts in some cases. However, the extent to which repetitive motor behaviors influence Fitbit-derived step counts in adults with ASD remains largely unknown. To our knowledge, there is limited evidence regarding the validity of Fitbit-derived step counts in adults with ASD, particularly among individuals who exhibit prominent repetitive motor behaviors. While prior studies have successfully used Fitbit devices to monitor physical activity and evaluate physical activity interventions in adults with ASD, these studies were not designed to establish the criterion validity of step-count measurements in this population [5].

The purpose of our study was to examine the construct validity and test-retest reliability of remotely administered physical function assessments in adults with ASD. Consistent with our analytic framework, Fitbit-derived step counts were included as one of several indicators of physical activity and functioning, alongside measures of lower-extremity function, fall risk, and comorbidity burden, to evaluate the construct validity of patient-reported physical function. Fitbit step counts were not intended to serve as a criterion standard for physical function assessment.

We agree that future studies comparing Fitbit-derived step counts with criterion measures such as direct observation, video-coded step counts, or research-grade accelerometry in adults with ASD would be valuable. Research-grade devices that provide access to raw acceleration signals may allow investigators to better distinguish ambulatory activity from stereotypic movements. However, these measures involve greater cost, analytic complexity, and participant burden. Consumer wearables such as Fitbit devices remain a viable option for large-scale and remote studies because they provide a practical, scalable, and objective estimate of physical activity. Establishing their validity and limitations in adults with ASD is therefore an important area for future research. However, such validation was beyond the scope of the current pilot study and protocol.

We appreciate the authors for highlighting this important issue. Continued research on the validity of wearable activity-monitoring technologies in adults with ASD will help strengthen the interpretation and application of physical activity metrics in both research and clinical practice.

## Abbreviations

ASD: autism spectrum disorder
